# Prospective Study of the Phenotypic and Mutational Spectrum of Ocular Albinism and Oculocutaneous Albinism

**DOI:** 10.3390/genes12040508

**Published:** 2021-03-30

**Authors:** Hwei Wuen Chan, Elena R. Schiff, Vijay K. Tailor, Samantha Malka, Magella M. Neveu, Maria Theodorou, Mariya Moosajee

**Affiliations:** 1 Moorfields Eye Hospital NHS Foundation Trust, London EC1V 2PD, UK; hwei_wuen_chan@nuhs.edu.sg (H.W.C.); e.schiff@ucl.ac.uk (E.R.S.); vijay.tailor.09@ucl.ac.uk (V.K.T.); samantha.malka@nhs.net (S.M.); magella.neveu@nhs.net (M.M.N.); mtheodorou@nhs.net (M.T.); 2 Institute of Ophthalmology, University College London, London EC1V 9EL, UK; 3 Department of Ophthalmology, National University Singapore, Singapore S118177, Singapore; 4 Experimental Psychology, University College London, London WC1H 0AP, UK; 5 Great Ormond Street Hospital for Children NHS Foundation Trust, London WC1N 3JH, UK; 6 The Francis Crick Institute, London NW1 1AT, UK

**Keywords:** albinism, foveal hypoplasia, nystagmus, chiasmal misrouting, oculocutaneous albinism, ocular albinism, Hermansky–Pudlak syndrome

## Abstract

Albinism encompasses a group of hereditary disorders characterized by reduced or absent ocular pigment and variable skin and/or hair involvement, with syndromic forms such as Hermansky–Pudlak syndrome and Chédiak–Higashi syndrome. Autosomal recessive oculocutaneous albinism (OCA) is phenotypically and genetically heterogenous (associated with seven genes). X-linked ocular albinism (OA) is associated with only one gene, *GPR143*. We report the clinical and genetic outcomes of 44 patients, from 40 unrelated families of diverse ethnicities, with query albinism presenting to the ocular genetics service at Moorfields Eye Hospital NHS Foundation Trust between November 2017 and October 2019. Thirty-six were children (≤ 16 years) with a median age of 31 months (range 2–186), and eight adults with a median age of 33 years (range 17–39); 52.3% (*n* = 23) were male. Genetic testing using whole genome sequencing (WGS, *n* = 9) or a targeted gene panel (*n* = 31) gave an overall diagnostic rate of 42.5% (44.4% (4/9) with WGS and 41.9% (13/31) with panel testing). Seventeen families had confirmed mutations in *TYR* (*n* = 9), *OCA2*, (*n* = 4), *HPS1* (*n* = 1), *HPS3* (*n* = 1), *HPS6* (*n* = 1), and *GPR143* (*n* = 1). Molecular diagnosis of albinism remains challenging due to factors such as missing heritability. Differential diagnoses must include *SLC38A8*-associated foveal hypoplasia and syndromic forms of albinism.

## 1. Introduction

Albinism encompasses a group of rare genetic disorders, frequently but not always associated with impaired melanosome maturation, melanin pigment synthesis, and distribution in melanocytes. Melanocytes are neural crest cells, which can be categorized into cutaneous (hair and skin) or extracutaneous (eye and cochlea). In mammalian eyes, the posterior epithelial surface of the ciliary body (pars plana), sphincter, and dilator muscles of the iris and retinal pigment epithelium (RPE) are derived from the neuroectoderm. The mesenchyme forms the connective tissue and blood vessels of the iris and ciliary body stroma. The choroid and uveal melanocytes as well as the hair and skin melanocytes are neural crest derived. During embryonic development the RPE is the first pigmented tissue to be observed due to early expression of melanogenic genes [1,2]. Melanin exists in two forms: eumelanin (black or dark brown) and pheomelanin (red or yellow). Melanin production is initiated by the conversion of tyrosine to L-DOPA by interacting with tyrosinase. The biosynthetic pathways for the two pigments diverge downstream of L-DOPA, determined by the signaling activity of the melanocortin-1 receptor (MC1R), a member of a subgroup of class A G-protein-coupled receptors, responsible for natural pigment variations in humans [3]. Eumelanogenesis involves two melanogenic enzymes, tyrosinase-related protein 1 (TYRP1) and dopachrome tautomerase (DCT), while pheomelanogenesis is cysteine dependent. Melanin is deposited within melanosomes. Variations in melanosome composition and structure determine the degree of pigmentation of the eyes, hair, and skin. In the eye, normal melanin metabolism is important in retinal pigment histogenesis, retinal ganglion cell metabolism, the organization and trajectory/projection of retinal-fugal fibers [4,5,6]. Impaired melanin biosynthesis, therefore, disrupts the delicate embryological processes of retinal differentiation and optic chiasm decussation, culminating in nystagmus and reduced visual acuity at birth. Ophthalmic manifestations of absent or reduced melanin pigment also include iris hypopigmentation with a transillumination defect, foveal hypoplasia, hypopigmented fundus, and the hallmark finding of chiasmal misrouting (increased number of axons crossing the optic chiasm to innervate the contralateral cortex) on flash and pattern visual evoked potential (VEP).

Oculocutaneous albinism (OCA) is an autosomal recessive disorder affecting all pigmented tissues in the eyes, hair, and skin to varying degrees. The estimated prevalence of OCA is 1 in 20,000 individuals [7]. Due to founder effects such as in America, Africa, India, and Japan, the global incidence and distribution of OCA is highly variable between populations. OCA comprises eight clinical subtypes, OCA1-8, with mutations in seven different genes encoding either enzymes or membrane transporter proteins that are involved in melanin synthesis and accumulation of tyrosine. OCA1, caused by mutations in tyrosinase (*TYR*), can be further categorized into OCA type IA (OMIM #203100), characterized by complete lack of tyrosinase activity due to production of an inactive enzyme (“tyrosinase negative”, complete OCA), and OCA type IB (OMIM #606952), characterized by reduced activity of tyrosinase (“tyrosinase positive”, partial OCA). OCA1 is the most common subtype in the white Caucasian population with an estimated prevalence of 1 in 40,000, accounting for approximately 50% of cases worldwide [8]. OCA2 (OMIM #203200) caused by mutations in the *OCA* gene, formerly known as the “*P*” gene, is the most common form of albinism worldwide; the estimated prevalence is 1 in 10,000 in the African American population, 1 in 30,000 in Caucasians, and closer to 1 in 3,900 in southern parts of Africa [9,10,11,12]. OCA3 (OMIM #203290) has an incidence rate of 1 in 8500 amongst patients of African ethnicity and is less severe compared to the other OCA subtypes [13,14]. It is caused by mutations in tyrosine-related protein 1 (*TYRP1*), which encodes an enzyme involved in the downstream biosynthesis of eumelanin from tyrosine [15]. Mutation in *TYRP1* is associated with the early degradation of tyrosinase and late maturation of melanosomes [16,17]. OCA4 (OMIM #606574) is caused by mutations in *SLC45A2*, a membrane-associated transporter protein (MATP) gene, and though generally uncommon (estimated prevalence 1 in 100,000), it is more prevalent in the Japanese population, accounting for 24% of the OCA cases [18]. OCA5 (OMIM #615312) was mapped to a locus on 4q24, but the specific gene underlying the pathogenesis remains to be determined [19,20]. OCA6 (OMIM #113750) results from mutations in a protein transporter gene *SLC24A5* also involved in melanin synthesis. OCA7 (OMIM #615179) is associated with mutations in the *LRMDA* or *c10orf11* gene, which encodes the leucine-rich melanocyte differentiation-associated protein. OCA8 (OMIM #619165) is caused by mutation in the dopachrome tautomerase (*DCT*) gene, which catalyzes dopachrome to produce dihydroxycarboxylic acid in the eumelanin synthesis pathway [21]. OCA5-8 are ultra-rare.

Ocular albinism (OA, OMIM #300500) is inherited as an X-linked disorder primarily affecting the eyes with relatively normal hair and skin pigmentation. The estimated prevalence is 1 in 60,000 males [7]. OA is caused by mutations in the G-protein coupled receptor 143 (*GPR143*) gene, also known as OA1, expressed in melanocytes. *GPR143* traffics to the membrane of melanosomes and regulates transcription of melanosome genes as well as melanosome size by disrupting the delivery of melanin-related protein (MRP) to mature melanosomes [22,23]. Impaired GPR143 signaling causes alterations in the melanosome morphology, resulting in the formation of enlarged melanosomes (“macromelanosomes”), disruption in melanosome motility, and overall reduction in melanosome numbers in the melanocytes and RPE [24,25]. *GPR143* is located on chromosome Xp22. Female carriers of *GPR143* mutations exhibit mosaicism due to different degrees of lyonization or random X chromosome inactivation, with the most characteristic finding being bright radial reflex (tapetal reflex) giving rise to a ‘mud-splatter’ appearance on fundus autofluorescence (FAF) imaging [26,27]. 

Syndromic OCA includes Hermansky–Pudlak syndrome (defective platelet adhesion causing easy bruising and severe bleeding), Chédiak–Higashi syndrome (immune deficiency with increased risk of infection, easy bruising, and bleeding due to thrombocytopaenia), and Griscelli syndrome. These are autosomal recessive conditions caused by one or more genes associated with lysosomal protein trafficking. Chédiak–Higashi syndrome (OMIM #214500) is caused by homozygous or compound heterozygous mutation in the gene *LYST*. Three types of Griscelli syndrome (GS) have been reported. GS1 (OMIM #214450) is caused by homozygous mutation in the gene *MYO5A*, resulting in hypomelanosis, primary neurological impairment without immunological impairment, and haemophagocytic syndrome. GS2 (OMIM #607624) is caused by mutation in *RAB27A* and is associated with immune impairment. GS3 (OMIM #609227) can be caused by mutation in *MLPH* or *MYO5A*; it is characterized by hypomelanosis without neurological or immune impairment.

Hermansky–Pudlak syndrome (HPS) is a rare, autosomal recessive disorder of impaired lysosomal related organelles (LROs) synthesis, with an estimated global incidence of one to nine per 1,000,000 individuals (available online: www.orpha.net; accessed on 28 December 2020). The prevalence of each subtype may vary between populations owing to founder effects. HPS comprises a group of 11 multisystem disorders, HPS1-11, characterized by OCA and a combination of bleeding diathesis (secondary to impaired platelet aggregation), immunodeficiency (neutropaenia), pulmonary fibrosis, and/or inflammatory bowel disease. The causative genes for HPS1-11: HPS1 (*HPS1*)*,* HPS2 (*AP3B1*)*,* HPS3 (*HPS3*), HPS4 (*HPS4*), HPS5 (*HPS5*), HPS6 (*HPS6*), HPS7 (*DTNBP1* or *BLOC1S8*), HPS8 (*BLOC1S3*), HPS9 (*BLOC1S6*), HPS10 (*AP3D1*), and the recently reported HPS11 (*BLOC1S5*) [28,29]. Though generally rare, it is important to exclude syndromic OCA as certain systemic associations may present later in life and carry significant morbidity and mortality.

Although OA and OCA are distinct genetic entities, OCA is phenotypically heterogenous and may overlap with OA. Distinguishing between the two based on clinical phenotype alone may be challenging. It is important to note that subtle hypopigmentation, especially in individuals with lightly-complected ancestry, may not be reliably appreciated and coded. Furthermore, considerable phenotypic overlap between albinism and other conditions such as foveal hypoplasia 2 (FVH2, OMIM #609218, also known as foveal hypoplasia 2, optic nerve decussation defects, and anterior segment dysgenesis (FHONDA) syndrome) caused by *SLC38A8* mutations may also confound the diagnosis. Therefore, molecular confirmation should be sought and correlated with clinical findings before establishing a definitive diagnosis. The purpose of this study is to describe the phenotypic and genotypic spectrum of a cohort of 44 consecutive patients presenting to the ocular genetics service with suspected albinism.

## 2. Materials and Methods

This was a prospective cohort study of consecutive nystagmus patients with suspected albinism presenting to the ocular genetic service at Moorfields Eye Hospital NHS Foundation Trust (MEH), London, United Kingdom, between November 2017 and October 2019. Patients who satisfied one of the following inclusion criteria were recruited; (i) positive family history of albinism with or without molecular confirmation of the affected family member(s), (ii) nystagmus with hypopigmentation of the fundus, hair and/or skin, (iii) nystagmus and foveal hypoplasia and/or intracranial chiasmal misrouting.

Data collected included medical history, family history, pedigree, hair and skin color, best corrected visual acuity (BCVA) and the presence of nystagmus, iris transillumination defect on slit lamp biomicroscopy as well as foveal reflex and fundus hypopigmentation on fundoscopy. VA assessment for all preverbal children up to 36 months of age was performed using Cardiff cards, for all other patients, BCVA was recorded in LogMAR (and converted to this if given in Snellen for statistical analysis). Visual acuity of 1/60 or counting fingers, hand movements, light perception, and no perception of light was recorded as 1.98, 2.28, 2.6, and 3.0, respectively. Anterior segment imaging of the iris was performed using the Haag-Streit slit lamp camera (Haag-Streit Holdings AG, Köniz, Switzerland). Ultra-widefield (UWF) pseudocolor and autofluorescence (AF) fundus imaging were acquired using the Optos^®^ California (Optos plc, Dunfermline, UK) and spectral-domain optical coherence tomography (SDOCT) was performed using the OCT SPECTRALIS^®^ (Heidelberg Engineering GmbH, Heidelberg, Germany). Chiasmal misrouting was confirmed either with multichannel flash VEP alone (for younger paediatric patients) or a combination of both flash and pattern VEP were performed at the Department of Electrophysiology, MEH. 

DNA samples extracted from peripheral blood with informed consent was used for genetic testing. Genetic testing was performed in the clinical and research setting, using the albinism and nystagmus targeted gene panel (Oculome; available online: http://www.labs.gosh.nhs.uk/media/764794/oculome_v8.pdf; accessed on 28 December 2020) consisting of 30 genes (*AP3B1, BLOC1S3, BLOC1S6, C10ORF11, CACNA1A, CACNA1F, CASK, DTNBP1, FRMD7, GPR143, HPS1, HPS3, HPS4, HPS5, HPS6, LYST, MANBA, MITF, MLPH, MYO5A, OCA2, PAX6, RAB27A, SACS, SETX, SLC24A5, SLC45A2, TULP1, TYR, TYRP1*) through the Rare & Inherited Disease Genomic Laboratory at Great Ormond Street Hospital (London, UK) and whole genome sequencing (WGS) as part of the UK Genomics England 100,000 Genomes Project, where the results were reviewed by a multidisciplinary team to confirm variant pathogenicity, prevalence in publicly available genome databases, the clinical phenotype and mode of inheritance, before the molecular diagnosis was established [30,31,32]. Genomic DNA was processed using an Illumina TruSeq DNA PCR-Free Sample Preparation kit (Illumina Inc., San Diego, CA, USA) and sequenced using an Illumina HiSeq X Ten high-throughput sequencing platform, generating minimum coverage of 15 X for >97% of the callable autosomal genome. Readings were aligned to either build GRCh37 or GRCH38 of the human genome using an Isaac aligner (Illumina Inc.). Single-nucleotide variants (SNVs) and indels (insertions or deletions) were identified using Platypus soft-ware (version 0.8.1; and annotated using Cellbase software (available online: https://github.com/opencb/cellbase; accessed on 28 December 2020). Variant filtering was performed using minor allele frequency (MAF) < 0.001 in publicly available and in-house data sets, predicted protein effect, and familial segregation. Surviving variants were prioritized using the Retinal disorders version 2.120 (available online: https://panelapp.genomicsengland.co.uk/panels/307/; accessed on 28 December 2020) and the Albinism or congenital nystagmus version 1.10 (available online: https://panelapp.genomicsengland.co.uk/panels/511/; accessed on 28 December 2020) virtual gene panels [33]. Patients seen between November 2017 to September 2018 were recruited into the 100,000 Genomes Project. Upon completion of recruitment to the 100,000 Genomes Project in 2018, all subsequent sequencing was performed using the targeted gene panel (Oculome). Affected siblings within the same family were all sequenced. Genetic samples of parents and/or additional family members were obtained where available. 

In silico tools (PolyPhen-2 [34] and SIFT [35]) were used to predict pathogenicity of the identified missense variants (Table 1). A missense variant was deemed pathogenic when predicted to be “probably damaging” by PolyPhen-2 and “deleterious” by SIFT. Frameshift, splice site, and nonsense mutations were all predicted to be deleterious as a result of nonsense mediated decay (NMD) or protein truncation.

This study had relevant local and national research ethics committee approvals (MEH and the Northwest London Research Ethics Committee) and adhered to the tenets of the Declaration of Helsinki. Patients and relatives gave written informed consent for participation in this study through either the Genetic Study of Inherited Eye Disease (REC reference 12/LO/0141) or Genomics England 100,000 Genomes project (REC reference 14/EE/1112).

## 3. Results

### 3.1. Clinical Findings

This prospective study identified 44 probands from 40 families, of which 47.7% (*n* = 21) were female and 52.3% (*n* = 23) were male. The study cohort comprised 36 children (≤ 16 years) with a median age of 31 months (range 2–186), and eight adults with a median age of 33 years (range 17–39). This cohort consisted of families with diverse ethnic origins, 11 (27.5%) White British, 8 (20.0%) South Asian, 5 (12.5%) mixed White and Black African, 5 (12.5%) White other, 4 (10.0%) African, 4 (10.0%) Black African, 2 (5.0%) Middle Eastern, and 1 (2.5%) mixed White and South Asian. One African family and 3 South Asian families were consanguineous (9%, *n* = 4/44). A positive family history was absent in 81.8% of probands (*n* = 36/44) with the inheritance pattern being unclear.

**Figure 1:**
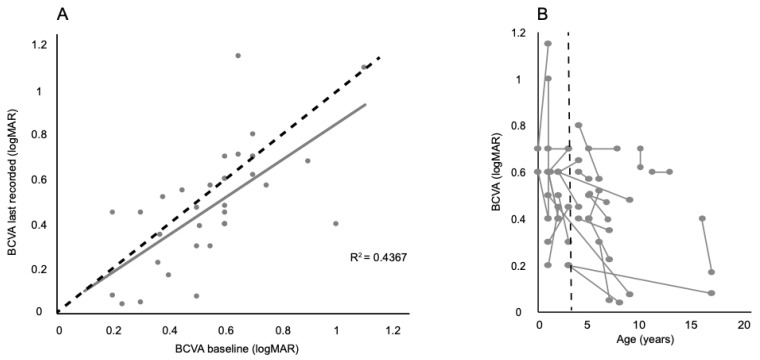
Best corrected visual acuity (BCVA) changes in albinism patients. (**A**) Correlation between the change in BCVA at baseline and most recent follow up. The black dashed line represents a ‘no change’ scenario, while the grey correlation line illustrates the subtle change seen in patients. (**B**) Demonstrates the change in BCVA of each paediatric patient at baseline and most recent vision as a function of age. The black dashed line represents the point at which more accurate visual acuity testing can be achieved in children 3 years old and upwards.

In the paediatric cohort, BCVA ranged from fixing and following to 1.2 logarithm of the minimal angle of resolution (LogMAR) with a median of 0.8 LogMAR. In the adult group, the range was 0.3 to 1.0 LogMAR with a median of 0.6 LogMAR. Correlation between the change in BCVA at baseline and most recent follow up visit demonstrated subtle change in BCVA over time (Figure 1A). The change in BCVA of each paediatric patient at baseline and most recent vision as a function of age is illustrated in Figure 1B. The average BCVA at baseline decreases from 0.6 to 0.48 logMAR for patients between 3–20 years of age. 

**Figure 2:**
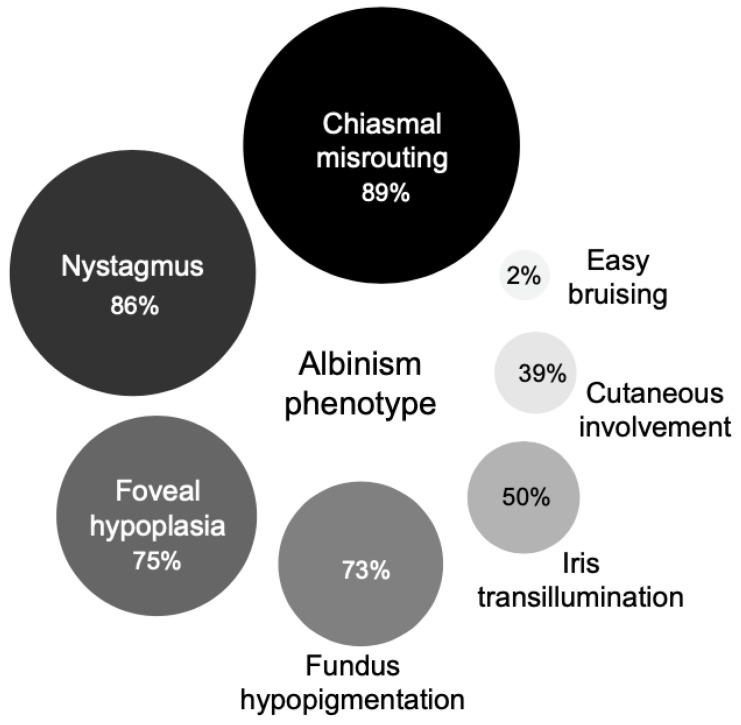
Overview of the phenotypic spectrum of 44 suspected albinism patients presenting to the ocular genetics service. For chiasmal misrouting, *n* = 37, 7 declined electrophysiology visual evoked potential (VEP) testing.

Of the cohort, 89.2% (*n* = 33/37, 2 were inconclusive, 2 had no chiasmal misrouting; 7 declined electrophysiology testing) had intracranial chiasmal misrouting, 86.4% (*n* = 38/44) had nystagmus, 75.0% (*n* = 33/44) had foveal hypoplasia, 72.7% (*n* = 32/44) had fundus hypopigmentation, 50.0% (*n* = 22/44) had iris transillumination defect, 38.6% (*n* = 17/44) had hair and skin (cutaneous) involvement, and 1 patient reported history of easy bruising (2.3%, *n* = 1/44) (Figure 2). The demographics and phenotypes are summarized in Table S1.

### 3.2. Genetic Sequencing Outcomes

Initial recruitment comprised 44 families, of which 12 underwent WGS and 32 families had targeted gene panel testing. WGS identified biallelic variants in *SLC38A8* in three unrelated families causing FVH2, all had foveal hypoplasia and intracranial chiasmal misrouting. Panel testing confirmed a *FRMD7* hemizygous mutation causing X-linked congenital nystagmus in one family with suspected albinism, where the VEP was inconclusive. All four families were excluded from subsequent analysis of albinism cases, but further clinical and genetic details are included under Section 3.3.5. Non-Albinism Cases.

**Figure 3:**
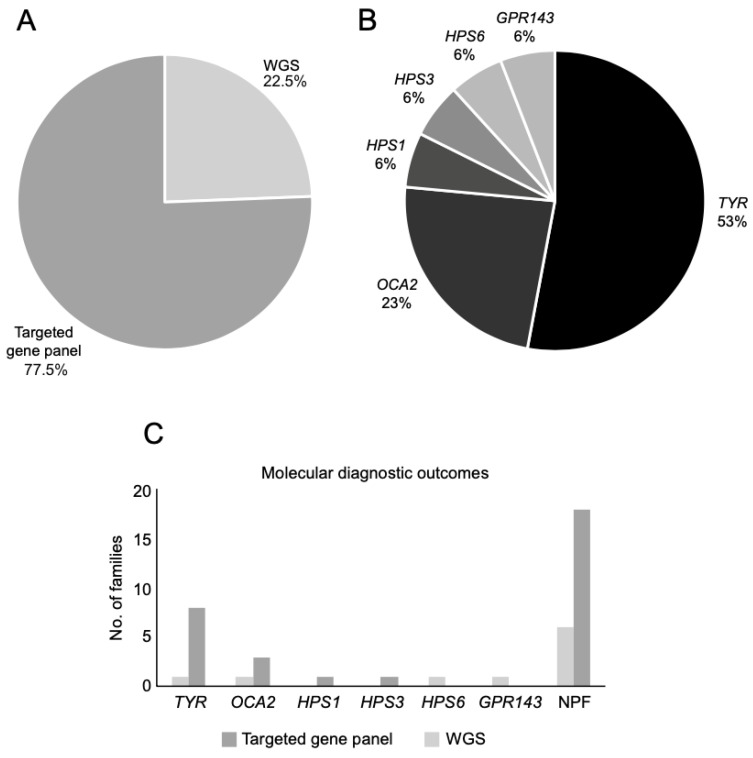
(**A**) Proportion of families who underwent whole genome sequencing (WGS) and targeted gene panel testing seen through the ocular genetics service between November 2017 and September 2019 at MEH (Moorfields Eye Hospital NHS Foundation Trust). (**B**) Mutational spectrum of families with positive molecular findings. (**C**) Molecular diagnostic outcomes for WGS and targeted panel sequencing for the entire cohort. NPF = no primary findings.

The overall diagnostic yield for albinism was 42.5%; 44.4% (4/9) with WGS, and 41.9% (13/31) with panel testing. Seventeen families had confirmed mutations (Table 1, Figure 3), of which 13 had OCA. OCA1 was the most common type with 69.2% (9/13) of families harboring *TYR* variants, followed by *OCA2* in 30.8% (4/13). Three families were diagnosed with Hermansky–Pudlak syndrome with pathogenic variants in either *HPS1* (6.0%; 1/17), *HPS3* (6.0%; 1/17) or *HPS6* (6.0%; 1/17). Only one family (6.0%; 1/17) had X-linked OA with a *GPR143* missense variant. Amongst the unsolved cases, a single pathogenic *TYR* variant was identified in one White British family (26649) with OCA1A and a single pathogenic *TYRP1* variant causing OCA3 in one Afro-Caribbean family (26948). Unsolved cases from the targeted panel testing will undergo WGS, and for those unsolved following WGS, further data mining will be undertaken to discover novel genes or noncoding variants. 

### 3.3. Genotype-Phenotype Correlation

#### 3.3.1. Oculocutaneous Albinism Type I (TYR)

**Figure 4:**
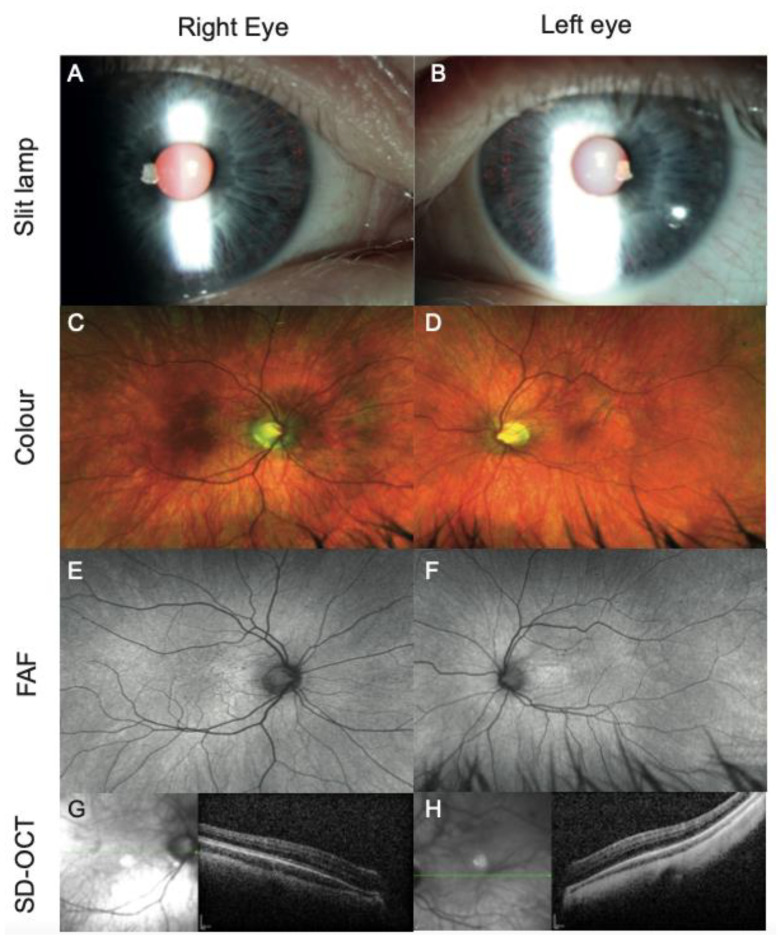
Iris and multimodal retinal imaging (right and left eye) of a patient (27079) with homozygous *TYR* mutation. (**A**,**B**) Iris transillumination defects with visualization of nasal lens equator; (**C**,**D**) ultra-widefield (UWF) pseudocolor showing hypopigmented fundi and prominent choroidal vessels; (**E**,**F**) UWF fundus autofluorescence (FAF) illustrating the absence of hypoautofluoresence at the fovea due to decreased luteal pigments, crossing of retinal vessels at the fovea, and prominent choroidal vessels can be appreciated; (**G**,**H**) grade 4 foveal hypoplasia on spectral-domain optical coherence tomography (SDOCT).

**Figure 5:**
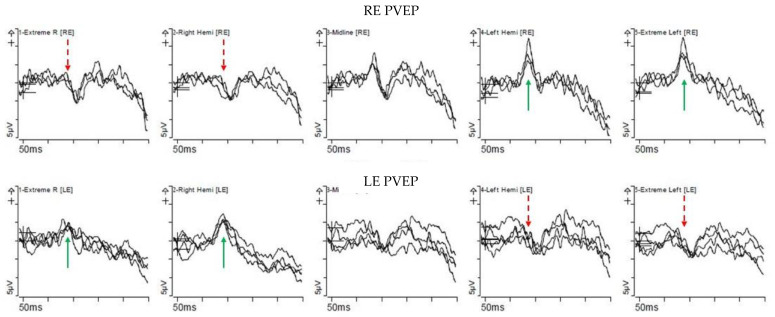
Pattern appearance VEPs (PVEP) from a patient (27079) with *TYR* mutation. PVEP responses on stimulating the right eye are displayed on the top row and those on stimulating the left eye are on the bottom row. Column 1 and 2 are responses from the right hemisphere, column 4 and 5 are responses from the left hemisphere, and column 3 is the combined midline response. PVEP shows contralateral predominance (turquoise arrow) when the opposite eye (dotted red arrow) is stimulated.

Of the nine families with molecularly confirmed *TYR* variants (Table 1), 4 had complete OCA1A and 5 had partial OCA1B (Table S1). The mean BCVA (in LogMAR) in the OCA1A group was 0.64 (range 0.2–1.0), compared to 0.6 (range 0.2–1.0) in the OCA1B group, with no significant difference between the groups. All patients exhibited clinical features of nystagmus, iris transillumination defect (TID), hypopigmented fundus, and foveal hypoplasia (Figure 4). Chiasmal misrouting was confirmed in all patients who underwent VEP testing (*n* = 3, 6 declined testing) (Figure 5). 

Of the *TYR* variants identified in this cohort, 11 were missense, 2 were splice-site and 1 was nonsense. The most commonly observed OCA1A variant in patients with OCA, c.1118C>A p.(Thr373Lys), was identified in the proband of a White British family (13332) with BCVA of 1.0 LogMAR and the full complement of albinism features. The same variant was also identified in an unsolved White British family (26649). This variant was reported to occur in approximately 30% of Northern European cases [8,36,37]. The proband of a consanguineous Pakistani family (26903) had BCVA of 1.0 LogMAR and all ocular features of albinism were present without obvious cutaneous involvement. She was homozygous for a loss-of-function *TYR* variant causing premature translational stop signal (c.832C>T, p.Arg278*). Several studies reported higher incidence of this causal *TYR* variant amongst South Asians (Indian, East Indian/West Bengal, Pakistani) due to consanguinity [38,39]. A recurring pair of hypomorphic alleles c.575C>A, p.(Ser192Tyr) and c.1205G>A, p.(Arg402Gln) was seen across 3 unrelated White British families with OCA1B, occurring in trans with 3 different pathogenic missense variants. In this OCA1B subgroup, the mean BCVA (in LogMAR) was 0.74 (range 0.6–1.0), all probands had iris TID, foveal hypoplasia and fundus hypopigmentation, nystagmus was subtle in one patient, clearly present in the other two. All 3 probands were of fair complexion that is similar to their unaffected relatives. In a French study of 268 patients with OCA1, the p.(Arg402Gln) variant in trans was identified in 69 patients (25.7%) with variable but generally mild forms of albinism [40].

#### 3.3.2. Oculocutaneous Albinism Type II (OCA2)

Four families (two mixed White and Black African, one Middle Eastern, one Black African) had *OCA2* mutations, 50% had oculocutaneous involvement. The mean BCVA (in LogMAR) was 0.5 (range 0.2–1.2). All families had nystagmus and chiasmal misrouting. Other clinical findings of iris TID, foveal hypoplasia, and fundus hypopigmentation were more variable within this subgroup compared to OCA1. Seven pathogenic *OCA2* variants were identified: 4 missense, 1 in-frame deletion, 1 frameshift deletion, 1 non-coding (splice). The same missense variant c.1327G>A, p.(Val443Ile) was seen in 2 unrelated families occurring with a different pathogenic variant (1 deletion, 1 missense). Variant p.Val443Ile is the most common in northern European populations [41,42], but is rare in other populations (<1% in the Scandinavian population) [43]. It is associated with residual function of the P protein (encoded by *OCA2*) and development of cutaneous pigment with time in affected individuals [44]. Despite impaired or diminished eumelanin synthesis, this progressive increase in pigmentation and the propensity to tan is due to the relative preservation of pheomelanin [45,46]. In this study, all probands harboring this variant did not have obvious cutaneous involvement but displayed fundus hypopigmentation with a mean BCVA (in LogMAR) of 0.3 (range 0.2–0.4). Family 27321 with missense variant c.2228C>T, p.(Pro743Leu) and splice donor variant c.1182+1G>A displayed a more severe phenotype with 1.2 LogMAR vision and the full complement of albinism features. 

#### 3.3.3. Hermansky–Pudlak Syndrome (HPS)

Four patients from three unrelated families were identified with HPS. The mean BCVA (in LogMAR) of this group was 0.6 (range 0.3–0.8). Foveal hypoplasia, iris TID, and chiasmal misrouting were present in 100% (*n* = 3), nystagmus and fundus hypopigmentation in 66.7% (*n* = 2). The lack of clinically apparent nystagmus was associated with relatively better visual prognosis in the paediatric cohort [47]. This finding was consistent in our cohort where probands from family 25588 without nystagmus recorded vision (in LogMAR) of 0.3 versus 0.6 and 0.8, respectively in families 26677 and 26847 with nystagmus. Significant systemic findings included spontaneous bruising in the proband of family 26677 with homozygous mutations in *HPS1* c.972dup, p.(Met325Hisfs*128). All patients were referred to haematology for further investigation and management.

#### 3.3.4. Ocular Albinism (GPR143)

The proband of Ghanaian family 25806 is a 13-year-old boy with a family history of maternal great uncles having yellow hair and fair skin. The patient had dark skin, black hair and reduced vision 0.7 and 0.6 LogMAR in the right and left eye, respectively. Examination was significant for nystagmus, although he had previously undergone the Anderson-Kestenbaum procedure in Ghana. He was found to have foveal hypoplasia and VEP confirmed chiasmal misrouting. WGS identified a pathogenic hemizygous mutation in *GPR143*: c.11C>G, p.(Pro4Arg) confirming the diagnosis of ocular albinism (OA1). 

#### 3.3.5. Non-Albinism Cases

All probands with *SLC38A8*-associated foveal hypoplasia had evidence of nystagmus, foveal hypoplasia, and intracranial misrouting. Family 23089 proband had bilateral posterior embryotoxon, no signs of anterior segment dysgenesis were seen in the other two families (26237 and 26320). Families 23089 and 26320 were consanguineous, carrying homozygous nonsense mutations in *SLC38A8*: c.264C>G, p.Tyr88*. Family 26237 had compound heterozygous *SLC38A8* mutations, c.435G>A, p.Trp145*, and c.632+1G>A. Further clinical details of these families were published [48]. 

The proband of White British family 27561 with a hemizgous *FRMD7* variant was a 3-year-old male with unilateral (left) grade 1b foveal hypoplasia (using the Leicester Grading System) on OCT [49]. He had blonde hair and fair skin, with 0.4 LogMAR vision bilaterally, fine horizontal nystagmus, and hypopigmented fundi. Repeated VEPs consistently showed equivocal findings on the right and contralateral predominance on the left. The equivocal VEP findings were partially attributed to maturational factors, but albinism could not be excluded. Targeted gene panel confirmed a novel pathogenic missense variant in *FRMD7* c.790T>G, p.(Cys264Gly). Pathogenic *FRMD7* mutations are fully penetrant in males [50]. *FRMD7* regulates brain development and neuronal growth by promoting neurite elongation and is expressed in the developing neural retina [51]. In patients with *FRMD7* variants, OCT imaging has shown abnormal afferent system development with foveal hypoplasia [52].

## 4. Discussion

This prospective observational study of 44 consecutive patients, from 40 families, presenting with query albinism reported an overall diagnostic yield of 42.5% through WGS or targeted gene panel testing. We defined diagnostic yield as the percentage of individuals with a characteristic clinical phenotype receiving a molecular diagnosis (≥2 pathogenic or likely pathogenic variants in a gene linked with OCA or ≥1 definite or likely pathogenic variant in *GPR143* for OA). Copy number variations (CNV) may partially account for the lower rate of diagnosis in this study. The clinical exome (Oculome) does not search for CNVs nor have they yet been analyzed by the Genomics England analysis pipeline. Molecular diagnostic rates for albinism are highly variable, ranging from 56–91% in the literature [32,53,54]. Lasseaux et al. (2018) reported a diagnostic yield of 72% from a large French study of 990 probands with albinism [53]. More recently, Lenassi et al. solved 29 out of 32 (91%) preschool children with suspected albinism using a targeted panel of 18 (*n* = 30) or 26 (*n* = 1) or 40 (*n* = 1) genes with confirmed mutations in *TYR* (*n* = 18), *OCA2* (*n* = 7), *TYRP1* (*n* = 2), *HPS5* (*n* = 1), and *GPR143* (*n* = 1). The authors did not specify the reason for selecting different gene-specific panels and the patient demographic details (aside from age) were not presented [54]. The probands who underwent expanded gene panel testing harbored *OCA2* and *TYR* variants. The *OCA2* variant c.1327G>A, p.(Val443Ile) and three *TYR* variants c.1118C>A, p.(Thr373Lys), c.575C>A, p.(Ser192Tyr), and c.1205G>A, p.(Arg402Gln) were also identified in our cohort through targeted gene panel testing. Comparison of the gene panels in the Lennasi study versus that employed in this study revealed some differences, e.g., they excluded *CACNA1A, CASK, HPS1* (suboptimal coverage)*, MANBA, MITF, MLPH, MYO5A, RAB27A, SACS, SETX,* and *TULP1,* whereas our panel did not include *ACO2, ATOH7, HMX1, RTN4IP1, SIX6,* and *SLC4A11*. However, none of these genes were identified in the Lenassi cohort, which suggests that stringency in clinical inclusion criteria is likely the major factor for increased likelihood of a positive finding. 

In our study, the majority of the probands were referred with nystagmus as a predominant clinical feature with equivocal findings of foveal hypoplasia and cutaneous involvement; 75% had foveal hypoplasia, 73% had fundus hypopigmentation, and 39% had skin/hair involvement. Ethnic/genetic diversity of the study cohort and possible under-ascertainment of cutaneous hypopigmentation may account for smaller percentage of skin/hair involvement reported in this study. Thirty-seven patients underwent VEP testing (seven declined) and chiasmal misrouting was confirmed in 33 patients (89%). Six out of the seven patients who declined VEP testing, had confirmed mutations in the *TYR* gene and one remained unsolved. This highlighted the importance of detailed phenotyping in delineating patients with albinism versus infantile nystagmus. Our diverse patient ethnicity may have also contributed to the difference due to rare/unknown ethnic-specific variants. Of the unsolved cases in this cohort, 45.0% (*n* = 18/40) underwent targeted panel testing, these patients will be offered WGS to identify potential novel genes/variants or non-coding mutations that remain to be determined.

Of interest, a recent genome-wide association study (GWAS) to determine the polygenic risk score for glaucoma susceptibility and progression identified a significant signal for *TYR* [55]. Although the original design of this study did not include glaucoma in the data collection, retrospective review of the clinical findings did not identify glaucoma in this cohort.

Several genetic eye disorders falling under infantile nystagmus syndrome (INS) can masquerade as albinism with considerable phenotypic overlap such as dominant *PAX6*-related oculopathy (OMIM # 136520), *SLC38A8*-related FVH2 (OMIM #609218), and forms of congenital nystagmus caused by *FRMD7* (OMIM #310700) or *GPR143* (OMIM #300814) [48,56,57]. Diagnosing albinism based on clinical findings alone is inadequate, particularly in infants and young children, where investigations such as optical coherence tomography (OCT) to assess foveal hypoplasia and VEPs for chiasmal misrouting may be challenging due to fixation loss and compliance. Candidate genes for INS should be grouped together for testing this cohort of patients, which may not yet be included in conventional albinism gene panels for comprehensive screening of patients [32]. 

In this study, OCA was the most common disease entity with *TYR* being the most common gene identified. Approximately 480 mutations were identified throughout the *TYR* gene; up to 77% were missense mutations (67% OCA1A and 33% OCA1B), 15% deletions, and 3% insertions [58,59]. In this cohort, 80% of the OCA1 variants were missense mutations. In cases of missing heritability where only a single pathogenic *TYR* variant was identified, studies subsequently identified two hypomorphic alleles, c.575C>A, p.(Ser192Tyr) and c.1205G>A, p.(Arg402Gln) in combination, forming a tri-allelic genotype [10,60,61,62]. Human melanocytes with the p.(Arg402Gln) variant retain tyrosinase in the endoplasmic reticulum (ER) and is associated with moderate thermoinstability resulting in approximately a 75% reduction of enzymatic activity at 37 °C [63,64]. The p.(Ser192Tyr) variant was shown to have 60% of wild type activity [65]. Together, the p.(Ser192Tyr) and p.(Arg402Gln) alleles result in the reduction of tyrosinase activity from retention in the ER and the released tyrosinase only having 60% of wild type activity, lowering the tyrosinase activity to a pathogenic level. We identified compound heterozygous tri-alleleic genotype in three unrelated families with partial OCA, where the p.(Ser192Tyr) and p.(Arg402Gln) alleles were seen in trans with a rare pathogenic *TYR* variant (different variant in each family). Various studies reported compound heterozygous tri-alleleic genotype in *TYR* involving both rare (AF <5%) and common (AF 28–36%) functionally damaging variants, which are likely to be on trans alleles [61]. The hypomorphic *TYR* coding variants p.(Ser192Tyr) and p.(Arg402Gln) are part of a pathogenic haplotype GYGQ, thought to explain up to 15% of individuals with albinism [62]. These variants, when in cis with each other result in a mild phenotype both when in trans with a pathogenic *TYR* mutation, and when homozygous [61,63]. Less pronounced iris translucency was observed in these patients [66]. Albinism should because of this, be considered as a diagnosis in children with subnormal visual acuity and/or otherwise unexplained nystagmus [62].

Two families with likely OCA, remained unsolved through targeted gene panel testing due to the identification of just one pathogenic variant. Family 26649 with OCA1A had a missense variant in *TYR* c.1118C>A, p.(Thr373Lys), the White British male proband had 0.54 LogMAR vision and all OCA1A features. Cell culture studies of this variant demonstrated protein misfolding/degradation and retention of mutant tyrosinase in the endoplasmic reticulum accounting for the phenotype [37]. Family 26948 were non-consanguineous White African, with two affected siblings with possible OCA3 harboring a heterozygous deletion in *TYRP1* c.1103del; p.(Lys368Serfs*17). The siblings had blonde hair and fair complexion with good BCVA 0.1 LogMAR, and no nystagmus. Aside from chiasmal misrouting, there were no ophthalmic features of albinism. OCA3 is common in the African population and known to have a less severe phenotype compared to the other OCA subtypes. Therefore, the identified pathogenic variant (AF 0.004973 in African or African Americans in gnomAD) [67], and a possible hypomorphic allele that has not yet been identified may explain the mild phenotype. The patients with missing heritability, would benefit from WGS to identify a second pathogenic variant in the promoter or other regulatory regions [68], or possible mutations in undiscovered OCA genes [69,70]. Deeper analysis may uncover synergistic interactions between known genes [71,72], dominant mutations not recognized due to pigmentation or ethnic background [60,73], hypomorphic mutations in known OCA genes and unrecognized splicing mutations or large deletions.

Three families were found to have homozygous mutations in the HPS gene, only one reported a history of spontaneous bruising. Syndromic forms of albinism are of clinical concern as affected individuals, particularly young children, can be overlooked. Genetic testing for albinism should include genes that cause syndromic forms, as these have considerable health implications. Three families (7% of the cohort) had molecular confirmation of HPS. Our findings of syndromic forms of albinism were consistent with previously reported rates of three to five percent [53,54]. Platelet granules and endothelial storage granules (Weibel–Palade bodies) are members of lysosome-related organelles whose formation is regulated by HPS protein-associated complexes such as biogenesis of lysosome-related organelles complex (BLOC-1, -2, and -3).

Genotype-phenotype correlations exist in HPS, where individuals with deficiencies in the biogenesis of lysosome-related organelle complex (BLOC), e.g., BLOC-2 deficiency (caused by *HPS3*, *HPS5*, or *HPS6*) display milder symptoms (such as minimal iris transillumination, fundus hypopigmentation, cutaneous involvement, and visual acuity as good as 0.4 LogMAR) than those with BLOC-3 deficiency (caused by *HPS1* or *HPS4*) [74]. Importantly, in patients harboring *HPS1* and *HPS4* mutations, pulmonary fibrosis is highly penetrant and a leading cause of premature death in adulthood, typically around the fourth or fifth decades of life, secondary to respiratory failure [75,76]. Current evidence indicates that 100% of patients with *HPS1* develop pulmonary fibrosis [77]. We identified one patient (family 26677) with homozygous *HPS1* variants but at four years of age, only displayed oculocutaneous features of albinism. There were no clear phenotypic severity markers between any of the HPS families, except that the proband of family 26847 with *HPS3* variants reported easy bruising, which is likely linked to their older age. Hence, early identification of syndromic OCA and coordinating the appropriate multidisciplinary care team, including paediatricians, respiratory specialists, immunologists, and haematologists, is critical to minimize morbidity and mortality of patients.

The correlation of BCVA at presentation and most recent visit was analyzed and did not show significant change overtime. Accurate documentation of functional vision is often challenging in young children. In this study, more accurate visual assessment could be achieved in children of age three years and above. No clear difference in mean BCVA (in LogMAR) was observed between the OCA1A (0.64), OCA1B (0.60), OCA2 (0.50), and HPS (0.60) subgroups. Refractive errors are common in albinism with myopia, hypermetropia and high levels of with-the rule astigmatism being reported [78,79]. To what extent the reduced visual acuity reflects the underlying pathology, nystagmus, foveal cone density, and whether these are genetically determined and how these factors influence/arrest normal emmetropisation in albinism remains debatable. 

Although this study had a relatively large sample size for a rare disease cohort, increased numbers of molecularly diagnosed patients will permit more accurate genotype-phenotype analyses. Further limitations of this study include the use of two different sequencing approaches, where targeted gene panels restrict the choice of genes/regions screened and non-coding interrogation.

## 5. Conclusions

In conclusion, we describe the phenotype and genotype of a series of patients with albinism. Albinism, in particular OCA, is phenotypically and genetically heterogenous and mild phenotypes may be easily missed. We highlighted the importance of careful phenotyping to yield a higher molecular diagnostic outcome for definitive diagnosis. Establishing the genetic cause as early as possible has the potential to inform management, allow the definition of specific care pathways, improve the understanding of developmental eye diseases, and aid future therapeutic development. Nevertheless, a substantial proportion of albinism cases remain genetically undetermined and further research is required to understand the genetic aetiology. As more variants and genes become associated with albinism, the diagnostic yield of genetic testing in these patients is likely to improve.

## Figures and Tables

**Table 1:** Variant details and confirmed phenotype for solved families. ^1^ Hypomorphic alleles pathogenic when in trans with a pathogenic variant. Autosomal recessive (AR), X-linked recessive (XL), heterozygous (Het), homozygous (Hom), hemizygous (Hem), Ashkenazi Jew (AJ).

Gene (OMIM)	Phenotype (OMIM)	Inheritance	Family ID (Ethnicity) [Consanguinity]	Allele 1 Allele2	Variant type	Zygosity	ClinVar ID	gnomAD	Polyphen-2	SIFT	Pathogenicity ACMG Classification	ACMG Arguments
*TYR* **(606933)**	Albinism, oculocutaneous, type 1A; OCA1A (203100)	AR	26352 (Middle Eastern) [No]	c.996G>A, p.(Met332Ile)	Missense	Het	Absent	Absent	Probably damaging	Deleterious	4	PM1; PM2; PM5; PP3; PP5; BP1
								
				c.1037-7T>A	Splice	Het	99527	0.000861, 0.015 in AJs	NA	NA	4	PP5; PM2; BP4
			22151 (White British) [No]	c.1336G>A, p.(Gly446Ser)	Missense	Het	3801	0.00005327	Possibly damaging	Deleterious	5	PP5; PM2; PP3; BP1
								
				c.242C>T, p.(Pro81Leu)	Missense	Het	3772	0.00009205	Probably damaging	Deleterious	5	PP5; PM1; PM2; PP3; BP1
			23192-1; 23192-2; 23192-3 (South Asian) [No]	c.1037-7T>A	Splice	Hom	Absent	Absent	NA	NA	4	PP5; PM2; BP4
			13332 (White British) [No]	c.1118C>A p.(Thr373Lys)	Missense	Het	3774	0.0003541	Probably damaging	Deleterious	5	PP5; PM1; PM2; PP3; BP1
								
				c.1A>G p.(Met1Val)	Missense	Het	3807	Absent	Possibly damaging	Deleterious	5	PVS1; PM2; PP3; PP5
	Albinism, oculocutaneous, type IB; OCA1B (606952)	AR	26680 (White British) [No]	c.575C>A, p.(Ser192Tyr) ^1^	Missense	Het	3778	0.2502	Probably damaging (both variants)	Deleterious (both variants)	Hypomorphic alleles ^1^	See text
				c.1205G>A, p.(Arg402Gln) ^1^	Missense		3779	0.1765				
								
				c.242C>T, p.(Pro81Leu)	Missense	Het	3772	0.00009205	Probably damaging	Deleterious	5	PP5; PM1; PM2; PP3; BP1
			27560 (White British) [No]	c.1217C>T, p.(Pro406Leu)	Missense	Het	3777	0.003918	Probably damaging	Deleterious	5	PP5; PM1; PM2; PP3; BP1
								
				c.1291C>A, p.(Pro431Thr)	Missense	Het	Absent	0.000007986	Probably damaging	Deleterious	4	PM1; PM2; PP3; PP5; BP1
			26903 (South Asian) [Yes]	c.832C>T, p.(Arg278*)	Nonsense	Hom	99583	0.0001699, 0.001274 in S Asians	NA	NA	5	PP5; PVS1; PM2; PP3
			27079 (White British) [No]	c.661G>A, p.(Glu221Lys)	Missense	Het	212524	0.00000399	Possibly damaging	Deleterious	4	PM1; PM2; PP3; PP5; BP1
								
				c.575C>A, p.(Ser192Tyr) ^1^	Missense	Het	3778	0.2502	Probably damaging (both variants)	Deleterious (both variants)	Hypomorphic alleles ^1^	See text
				c.1205G>A, p.(Arg402Gln) ^1^	Missense		3779	0.1765				
			27430 (White British) [No]	c.823G>T, p.(Val275Phe)	Missense	Het	3773	0.00009916	Possibly damaging	Deleterious	5	PP5; PM2; PP3; BP1
								
				c.575C>A, p.(Ser192Tyr) ^1^	Missense	Het	3778	0.2502	Probably damaging (both variants)	Deleterious (both variants)	Hypomorphic alleles ^1^	See text
				c.1205G>A, p.(Arg402Gln) ^1^	Missense		3779	0.1765				
*OCA2* **(611409)**	Albinism, oculocutaneous, type II (203200)	AR	25578 (Mixed: White / African) [No]	c.619_636del, p.(Leu207_Leu212del)	Deletion	Het	372713	0.0008030 in African / African Americans	NA	NA	4	PP5; PM2; PM4; PP3
								
				c.1327G>A, p.(Val443Ile)	Missense	Het	955	0.003056	Probably damaging	Deleterious	4	PS3; PM3; PP3; PP4; PP5; BS2
			25246 (Middle Eastern) [No]	c.1286T>C, p.(Leu429Pro)	Missense	Het	627601	Absent	Probably damaging	Deleterious	4	PM1; PM2; PP3; PP5; BP1
								
				c.1327G>A, p.(Val443Ile)	Missense	Het	955	0.003056	Probably damaging	Deleterious	4	PS3; PM3; PP3; PP4; PP5; BS2
			26947 (Mixed: White / African) [No]	c.2177_2181del, p.(Val726Glyfs*13)	Deletion	Het	498226	Absent	NA	NA	5	PVS1; PM2; PP3; PP5
								
				c.1255C>T, p.(Arg419Trp)	Missense	Het	194160	0.0002659	Probably damaging	Deleterious	4	PM2; PP3; PP5; PM3
			27321 (Black African) [No]	c.2228C>T, p.(Pro743Leu)	Missense	Het	956	0.0001344	Probably damaging	Deleterious	5	PP5; PM2; PP3; BP1
								
				c.1182+1G>A	Splice	Het	436099	0.0000566	Probably damaging	Deleterious	5	PVS1; PM2; PP3; PP5
*HPS1* **(604982)**	Hermansky- Pudlak syndrome 1 (203300)	AR	26677 (South Asian) [No]	c.972dup, p.(Met325Hisfs*128)	Duplication	Hom	Absent	0.000262	NA	NA	5	PVS1; PM2; PP3; PP5
*HPS3* **(606118)**	Hermansky- Pudlak syndrome 3 (614072)	AR	26847 (South Asian) [Yes]	c.850del, p.(Arg284Glufs*57)	Deletion	Hom	664198	Absent	NA	NA	5	PVS1; PM2; PP3
*HPS6* **(607522)**	Hermansky- Pudlak syndrome 6 (614075)	AR	25888-1; 25888-2 (Mixed: White / African) [No]	c.1228_1252del, p.(Tyr410Valfs*9)	Deletion	Hom	Absent	Absent	NA	NA	5	PVS1; PM2; PP3
*GPR143* **(300808)**	Ocular albinism, type I, Nettleship-Falls type (300500)	XL	25806 (African) [No]	c.11C>G, p.(Pro4Arg)	Missense	Hemi	Absent	Absent	Probably damaging	Deleterious	4	PM2; PP2; PP3; PP4; PP5

## Supplementary Materials

The following are available online at https://www.mdpi.com/article/10.3390/genes12040508/s1, Table S1: Overview of the patient demographics and clinical phenotype.
