# Supplementary material for: Prospective Study of the Phenotypic and Mutational Spectrum of Ocular Albinism and Oculocutaneous Albinism
Source: Genes (Basel). 2021 Mar 30;12(4):508. doi: 10.3390/genes12040508 (PMC8065601; doi:10.3390/genes12040508)
Supplement: Supplementary file 1 [file genes-12-00508-s001.pdf]

Table S1. Overview of the patient demographics and clinical phenotype. Mutations shaded in grey are inconclusive.

Family history (FH), Transillumination defect (TID), Unaffected (U), Affected (A), Present (P), Absent (-), Inconclusive (I), Declined (D), Fixing & following (FF).

Family ID	Gender	FH	Ethnicity	Consanguinity	Hair & skin	VA	Nystagmus	Iris TID	Foveal hypoplasia	Fundus hypopigmentation	Chiasmal misrouting
**25544**	M	-	African	-	U	0.74	P	-	P	-	P
**25578**	F	-	Mixed: White / African	-	U	0.2	P	-	-	P	P
**25704**	M	-	Mixed: White / Asian	-	U	1.12	P	-	-	P	P
**25806**	M	P	African	-	U	0.7	P	-	P	-	P
**25888-1**	F	P	African	-	U	0.3	-	P	P	P	I
**25888-2**	F	P	African	-	U	0.3	-	P	P	P	P
**26015**	M	-	White other	-	U	0.78	P	-	-	-	P
**26065**	M	-	White other	-	U	0.1	P	-	-	P	P
**26261**	F	-	South Asian	-	U	0.58	P	-	P	-	P
**26352**	F	-	Middle Eastern	-	A	0.5	P	P	P	P	D
**26640**	F	-	South Asian	-	U	0.6	P	-	P	P	P
**26641**	F	-	White other	-	U	0.6	P	-	P	P	P
**26680**	M	-	White British	-	U	0.64	-	P	P	P	P
**26677**	M	-	South Asian	-	A	0.6	P	P	P	-	P
**25246**	F	P	Middle Eastern	-	U	0.4	P	-	P	P	P
**26649**	M	-	White British	-	A	0.54	P	P	P	P	P
**26847**	M	-	South Asian	P	U	0.8	P	P	P	P	P
**26876**	M	-	Mixed: White / African	-	U	0.6	P	P	P	P	P
**26905**	F	-	South Asian	-	A	FF	P	-	P	P	P
**26948-1**	F	P	Mixed: White / African	-	A	0.15	-	-	-	-	P
**26948-2**	F	P	Mixed: White / African	-	A	0.1	-	-	-	-	P
**26947**	F	-	Mixed: White / African	-	A	0.3	P	-	-	-	P
**26984**	F	-	Black African	-	U	0.7	P	-	P	-	P
**27099**	M	-	Black African	-	U	1.12	P	-	-	-	P
**27101**	M	-	Black African	-	U	0.44	P	-	-	-	P
**23192-1**	F	P	South Asian	-	A	FF	P	P	P	P	I
**23192-2**	F	P	South Asian	-	A	0.84	P	P	P	P	P
**23192-3**	M	P	South Asian	-	A	0.2	-	P	P	P	P
**27268**	M	-	White British	-	U	0.2	P	-	P	P	P
**27321**	M	P	Black African	-	A	1.2	P	P	P	P	P

**27432**	F	-	White other	-	U	0.26	P	-	-	P	-
**27431**	F	-	White other	-	A	0.4	P	P	P	P	P
**27455**	M	-	White British	-	U	0.38	P	-	P	P	D
**27451**	M	-	African	P	U	0.6	P	-	P	P	P
**27560**	M	-	White British	-	U	0.2	P	P	P	P	D
**27655**	M	-	South Asian	P	U	0.3	P	-	-	-	P
**26903**	F	P	South Asian	P	U	1.0	P	P	P	P	D
**26956**	F	-	White British	-	U	0.6	P	P	P	P	-
**27033**	F	-	White British	-	A	0.3	P	P	P	P	P
**27047**	M	-	Mixed: White / African	-	A	0.6	P	P	P	P	P
**27079**	M	-	White British	-	U	1.0	P	P	P	P	P
**13332**	M	-	White British	-	A	1.0	P	P	P	P	D
**27430**	F	-	White British	-	U	0.6	P	P	P	P	D
**22151**	M	P	White British	-	A	0.78	P	P	P	P	D
